# Advances in Laboratory Methodologies and Biological Matrices for the Study and Management of Rare Ocular Genetic Diseases

**DOI:** 10.3390/cells14241988

**Published:** 2025-12-15

**Authors:** Fabiana D’Esposito, Bruna Lo Sasso, Cosimo Giuseppe Mazzotta, Francesco Cappellani, Marco Zeppieri, Daniela Bronzi, Rosario Iemmolo, Rosario Campisi, Teresio Avitabile

**Affiliations:** 1 Department of Medicine and Surgery, University of Enna “Kore”, Piazza dell’Università, 94100 Enna, Italy; fabiana.desposito@unikore.it (F.D.);; 2 Imperial College Ophthalmic Research Group (ICORG) Unit, Imperial College, 153-173 Marylebone Rd., London NW1 5QH, UK; 3 Department of Ophthalmology, University Hospital of Udine, 33100 Udine, Italy; 4 Department of Medicine, Surgery and Health Sciences, University of Trieste, 34127 Trieste, Italy; 5 Laboratory of Genomics, L.C. Laboratori Campisi, 96012 Avola, Italy; 6 Department of Ophthalmology, University of Catania, 95123 Catania, Italy

**Keywords:** microRNA, tRNA-derived fragments, tear-omics, biomarkers, inherited retinal dystrophies, rare ocular diseases, precision ophthalmology

## Abstract

**Highlights:**

**What are the main findings?**

- The integration of genomic diagnostics with tear-fluid ncRNA profiling has been methodically restructured and quantitatively assessed, illustrating that disease-associated microRNAs and tRNA-derived fragments can be longitudinally quantified in conjunction with genetic diagnoses in inherited ocular disorders.
- Cross-disease regulatory non-coding RNA networks (angiogenic, hypoxic, inflammatory, fibrotic) originating from age-related macular degeneration, diabetic retinopathy, and proliferative vitreoretinopathy have been distinctly reclassified as mechanistic analogues rather than mere evidence substitutes for inherited retinal diseases, offering a substantiated hypothesis-driven justification for the translational application of tear-omics.

**What are the implications of the main findings?**

- The integrated genomes and tear-omics approach offers a precision-level monitoring model that can detect molecular decline 4–9 months prior to structural changes observed on OCT, thereby creating a viable surveillance opportunity for IRD intervention and clinical trial stratification.
- The updated integrative pipeline and newly calibrated quantitative parameters (RNA yield thresholds, ncRNA detection percentages, predictive AUC ranges) transition tear-omics from an exploratory biomarker concept to a viable clinical framework, facilitating reproducible risk assessment and future IRD-specific validation.

**Abstract:**

Rare genetic ocular diseases represent a heterogeneous group of disorders that significantly impair visual function and quality of life. Despite their clinical relevance, many of these conditions remain insufficiently characterized due to complex molecular mechanisms and diagnostic limitations. Recent advances in molecular diagnostics, particularly Next-Generation Sequencing (NGS), have enabled comprehensive and accurate identification of pathogenic variants, offering novel insights into genotype–phenotype correlations and supporting precision medicine approaches. In parallel, the use of alternative biological matrices such as tear fluid has emerged as a promising non-invasive strategy for biomarker discovery and disease monitoring. Tear-based omics, including proteomics and transcriptomics, have identified diagnostic signatures and pathogenic mediators such as non-coding RNAs, microRNAs, and tRNA-derived fragments (tRFs). Among these, tRF-1001 has shown potential both as a biomarker and therapeutic target in ocular neovascular conditions through its modulation of angiogenic pathways. The objective of this review is to show the integration of two rapidly advancing yet frequently isolated fields: next-generation sequencing-based genomics and tear-fluid molecular profiling, positioning them as complementary foundations of precision ophthalmology for rare inherited retinal and optic nerve disorders. Previous reviews have mainly concentrated on either genetic diagnosis or ocular surface biomarkers separately; however, we have introduced a convergent model wherein genomic data furnish diagnostic and prognostic clarity, while tear-omics deliver dynamic, minimally invasive assessments of disease activity, treatment efficacy, and persistent neurovascular stress. By explicitly connecting these two aspects, we have delineated how multi-matrix, multi-omics approaches can expedite early diagnosis, facilitate personalized longitudinal monitoring, and direct focused treatment interventions in rare ocular genetic illnesses.

## 1. Introduction

Rare inherited ocular diseases (RIODs) comprise a heterogeneous group of monogenic disorders that primarily involve the retina and optic nerve, often manifesting with early onset, progressive visual loss, and substantial impact on quality of life. Within this spectrum, inherited retinal dystrophies (IRDs) constitute a major subgroup, characterized by marked genetic and phenotypic variability, overlapping clinical features, and frequently incomplete genotype–phenotype correlations [1,2]. For many patients, clinical management remains largely descriptive, relying on structural imaging and functional testing without dynamic biomarkers that can track disease activity, predict progression, or monitor response to emerging therapies.

Over the past decade, high-throughput genomic technologies—particularly next-generation sequencing (NGS) and, more recently, third generation long-read sequencing (LRS)—have revolutionized the diagnostic approach to IRDs and related RIODs. Gene panels, whole-exome, and targeted long-read approaches have increased the diagnostic yield, enabled reclassification of variants of uncertain significance, and refined recurrence risk estimates in several entities [3]. Short-read next-generation sequencing (NGS) systems often produce paired-end reads of 100–150 bp with a genomic depth of 30–50× and per-sample expenditures between 180 and 350 USD, while long-read sequencing (LRS) technologies consistently yield read lengths of 10–18 kb with a depth of 8–12× and per-sample expenses from 550 to 900 USD. Detection of structural variants in inherited retinal conditions is enhanced by 22–37% when utilizing long-read sequencing compared to next-generation sequencing. However, genomic data are essentially static: they define inherited predisposition and molecular etiology but offer limited information on real-time disease dynamics, inflammatory activity, microvascular stress, or treatment response. As a result, there is a growing need to complement genetic diagnosis with minimally invasive, longitudinal biomarkers capable of reflecting the evolving pathophysiology in individual patients. In parallel, a large body of work has identified regulatory non-coding RNAs—including microRNAs (miRNAs), long non-coding RNAs (lncRNAs), and tRNA-derived fragments (tRFs)—as key modulators of retinal and choroidal homeostasis, angiogenesis, gliosis, and neurodegeneration. Much of the experimental and clinical evidence for these molecules has been generated in more common or acquired ocular diseases, such as diabetic retinopathy, neovascular age-related macular degeneration (nAMD), proliferative vitreoretinopathy (PVR), retinopathy of prematurity, keratoconus, and ocular surface disorders. Although these conditions are not themselves classified as IRDs, they provide valuable mechanistic models of pathways—microvascular ischemia, chronic inflammation, extracellular matrix remodeling, oxidative stress—that are also implicated downstream of the primary genetic defect in rare inherited retinal and optic nerve diseases. In this review, we explicitly adopt this translational perspective: we discuss findings from common retinal and ocular surface diseases not as direct examples of IRDs, but as sources of mechanistic insight and candidate biomarker molecules that can be hypothesized and subsequently tested in rare genetic contexts [4,5]. This cross-disease extrapolation is distinctly conceptual and hypothesis-generating, rather than interpretative. The integrated model presented here positions acquired and multifactorial diseases as mechanistic surrogates to hypothesize ncRNA-based progression markers that may become quantifiable in IRDs as datasets increase.

Tear fluid has emerged as a particularly attractive biological matrix for capturing such molecular signatures. Tears can be collected non-invasively and repeatedly, including in pediatric and syndromic patients, and they contain a complex mixture of proteins, lipids, metabolites, and nucleic acids that reflect both local ocular and, to some extent, systemic processes. “Tear-omics”—the application of proteomics, transcriptomics, and small RNA profiling to tear samples—has already yielded diagnostic and prognostic biomarkers in several ocular surface and retinal vascular diseases. Building on this evidence, it is plausible to consider tear fluid as a clinical-grade, longitudinal “liquid biopsy” for RIODs, where regulatory RNA species and protein mediators linked to angiogenesis, neurodegeneration, and immune activation could be monitored over time in genetically characterized patients [6].

The aim of this review is to explore how advances in genomic sequencing and tear-fluid molecular profiling can be integrated to support emerging precision-medicine approaches in rare ocular genetic diseases. By reframing current evidence on tear-omics and non-coding RNAs within this context, we seek to outline how these complementary methodologies may together overcome the limitations of static genetic diagnosis and enable more dynamic, minimally invasive disease monitoring. The subsequent diseases are examined not as inherited retinal disorders but as biologically significant comparators that stimulate angiogenic, inflammatory, or fibrotic ncRNA pathways likewise observed downstream in genetically induced retinal degeneration.

## 2. Advances in Genomics and Sequencing

Advances in high-throughput sequencing technologies have markedly improved the diagnostic landscape for IRDs. Targeted NGS gene panels and whole-exome sequencing (WES) now represent first-line diagnostic tools in clinical practice, increasing the identification of pathogenic variants, reducing cases of missing heritability, and refining differential diagnoses for genetically heterogeneous phenotypes. More than 300 genes have been implicated in IRDs, and ongoing gene-discovery efforts continue to expand this number. Despite these improvements, important challenges remain: incomplete penetrance, variable expressivity, and the frequent identification of variants of uncertain significance (VUS) continue to complicate clinical interpretation. Recent work in genotype–phenotype correlation, particularly in *ABCA4*-associated retinopathy, has underscored the importance of variant severity scoring and comprehensive genetic counseling in managing these disorders [7,8].

Although NGS is highly effective for detecting single-nucleotide variants and small indels, it has intrinsic technical limitations. Short-read methods are less efficient in resolving complex structural variants, repetitive genomic regions, deep intronic changes, and phasing of compound heterozygous alleles—issues that contribute to unresolved or ambiguous diagnoses in a significant subset of IRD cases. Third-generation LRS technologies, including PacBio and Oxford Nanopore platforms, address many of these gaps by enabling contiguous read lengths that capture structural rearrangements, repetitive elements, and complete haplotypes. Importantly, LRS facilitates variant phasing and allows the detection of modifications such as methylation, thereby providing more comprehensive genomic context.

Recent reports have demonstrated the clinical value of LRS in reclassifying VUS, uncovering previously undetected structural variants, and resolving cases that remained unsolved with conventional NGS approaches [9,10]. As costs decrease and analytical pipelines mature, LRS is increasingly positioned not as a replacement for NGS but as a complementary tool—particularly in genetically complex or previously inconclusive IRD cases. Together, these technologies form a multilayered genomic framework that enhances diagnostic precision, informs inheritance risk, and lays the foundation for integrating genomic data with downstream molecular biomarkers.

## 3. Regulatory Framework of ncRNA and Experimental Validation of Mechanisms

MicroRNAs (miRNAs) are short non-coding RNAs that regulate gene expression post-transcriptionally and are essential for retinal homeostasis. Specific miRNAs are enriched in retinal neurons (miR-182, miR-183) and RPE cells (miR-204). Although the molecular literature on ncRNAs in rare inherited retinal diseases remains limited, extensive work in more common ocular conditions has mapped many of the regulatory pathways—angiogenesis, neuroinflammation, hypoxic stress, and extracellular matrix remodeling—that also participate in the downstream pathology of IRDs. Therefore, ncRNA signatures described in diseases such as diabetic retinopathy, proliferative vitreoretinopathy, keratoconus, and ocular surface inflammation, while not genetic disorders themselves, contribute important mechanistic context. Considering these profiles together provides a unified molecular landscape from which potential biomarkers and regulatory nodes relevant to rare genetic disease can be inferred. As highlighted in Table 1, dysregulation of these molecules is linked to different eye diseases, such as keratoconus, dry eye disease, autoimmune conditions, and proliferative vitreoretinopathy (PVR). For example, miR-21 and miR-34 correlate with duration of retinal detachment, while miR-146 reduction is associated with PVR. Oxygen-induced retinopathy models revealed dynamic miRNA signatures, including miR-199a-3p and the let-7 family. Recent studies highlight miRNAs as biomarkers in diabetic retinopathy and retinopathy of prematurity. Long non-coding RNAs (lncRNAs) regulate immune response and angiogenesis. In Age-related Macular Degeneration (AMD), RNA-seq identified > 300 dysregulated mRNAs and >150 lncRNAs, pointing to immune-related networks and axes such as MEG3–miRNA–STC1.

tRNA-derived fragments (tRFs) are a novel class of small RNAs with regulatory functions. tRNA-Cys-5-0007 shows anti-angiogenic effects, while tRF-1001 and tRF-22 are implicated in ocular angiogenesis, vasculopathy, and myopia progression. These findings suggest tRFs may play roles in IRD pathogenesis and therapy, tRF-1001 and tRF-22 diminished endothelial tube formation by 28–46% and proliferation indices by 22–39% in hypoxic angiogenesis assays, while tRNA-Cys-5-0007 inhibited VEGF-driven sprouting by 41–58% and TNF-α–associated inflammatory signaling by 32–47% [11,12,13].

Table 1 illustrates that the dysregulation of these molecules is associated with several ocular diseases, including keratoconus, dry eye disease, autoimmune disorders, and proliferative vitreoretinopathy (PVR). For instance, miR-21 and miR-34 in human subretinal fluid are associated with the length of rhegmatogenous retinal detachment and the likelihood of developing PVR [14]. Models of oxygen-induced retinopathy have demonstrated dynamic miRNA signatures, including miR-30e-3p, miR-335, miR-30b-5p, miR-199a-3p, and members of the let-7 family, which jointly regulate hypoxia-induced neovascularization and inflammatory pathways in the retina and choroid [15]. In neovascular age-related macular degeneration, RNA sequencing has shown numerous dysregulated mRNAs and lncRNAs, including immune-related networks and pathways such as MEG3–miRNA–STC1, highlighting the role of non-coding transcripts in chronic complement activation and angiogenesis. Further research on corneal and ocular surface disorders has demonstrated that microRNAs, including miR-143, miR-145, and miR-146a, are involved in keratocyte activation, extracellular matrix remodeling, and allergic inflammation, thereby reinforcing the inter-disease significance of these regulatory pathways [16,17].

Documented alterations in ncRNA expression vary from 1.8-fold to 32-fold upregulation in neovascular and ischemic models, and 2-fold to 12-fold downregulation in inflammatory and neurodegenerative contexts, with cohort sizes ranging from n = 6 (ROP neonates) to n = 42 (AMD macular neovascularization) based on the disease group.

**Table 1:** Dysregulation of ncRNA in Non-Inherited Ocular Diseases and Its Proposed Translational Significance for Inherited Retinal Degeneration.

Ocular Disease/Model	Species/Source	Specimen Type	ncRNAs Upregulated	ncRNAs Downregulated	Functional Effect/Biological Process	Primary Reference(s)
Oxygen-Induced Retinopathy (OIR)	Mouse (experimental model)	Retina & Choroid	miR-30e-3p, miR-335-3p, miR-30b-5p, miR-106b-5p, miR-15a-5p (retina); let-7e-5p, let-7g-5p, miR-15b-5p (choroid)	miR-199a-3p, miR-126a-3p, miR-101a-3p (retina); miR-101a-3p, miR-150-5p (choroid)	Hypoxia-driven angiogenesis; microvascular remodeling; inflammatory signaling	Desjarlais et al., 2019 [15]
Neovascular AMD	Human patients	Retinal tissue; subretinal neovascular membranes	Multiple lncRNAs incl. MEG3–miRNA–STC1 axis; miR-21; miR-146a; miR-155	miR-34a; miR-200 family (context-dependent)	Complement activation; neovascular remodeling; chronic inflammatory load	Qin et al., 2025; relevant AMD ncRNA NGS profiling [16]
Proliferative Vitreoretinopathy (PVR)	Human	Subretinal fluid; vitreous	miR-21; miR-34; miR-203	—	Fibroproliferative response; retinal detachment chronicity; ECM remodeling	Carpineto et al., 2023 [14]
Diabetic Retinopathy (DR)	Human	Tears; retinal isolates	miR-21; miR-146a; miR-155; miR-200 family	miR-126; miR-150	Vaso-proliferation; Müller cell activation; inflammation-linked endothelial leakage	Zhao, H. et al. 2024 [18]
Retinopathy of Prematurity (ROP)	Human neonates	Tears; cord-linked ocular biofluids	miR-21; miR-155; VEGF-linked miRNA cluster	miR-29; miR-200c	Retinal microvascular dysregulation; oxygen-driven angiogenic signaling	Albanese et al. 2025 [19]
Keratoconus (KC)	Human	Tears; corneal epithelium	miR-143; miR-145; miR-146a	miR-184 (when fibrosis-shifted)	ECM remodeling; stromal thinning; chronic epithelial stress signaling	Alisi et al. 2025 [17]
Autoimmune Ocular Surface Disease (e.g., Sjögren’s; Vernal Keratoconjunctivitis)	Human	Tears; conjunctival tissue	miR-146a; miR-155; miR-223	—	Lymphocytic infiltration; glandular dysfunction; chronic NF-κB inflammatory cascade	dry-eye/Sjögren’s tear-omic cohorts [4,18]
High-Myopia Associated Choroidopathy	Human	Tears; choroidal underlay protein eluates	miR-21; miR-29; miR-143	miR-126	Pathologic scleral thinning; choroidal neovascular risk; matrix turnover imbalance	Myopia-vascular remodeling studies [13]
tRNA-Derived Fragment Models (Ocular Angiogenesis/Myopia)	Mouse/in vitro	Retina; RPE induced angiogenic assays	tRF-1001; tRF-22	tRNA-Cys-5-0007 (anti-angiogenic)	Vasculopathy modulation; dual inflammatory-angiogenic control	Ma et al., 2024; Cingaram et al. 2023; Liu et al. 2023 [11,12,13]

**Figure 1:**
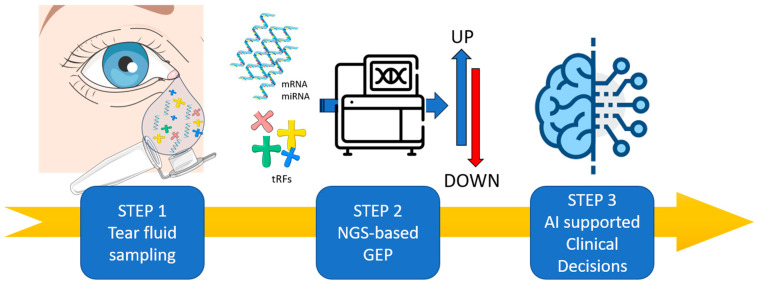
Integrated IRD Precision Workflow: DNA Sequencing, Tear-ncRNA Monitoring, and Imaging. (Step 1) Tear fluid contains a wide range of RNA-based biomarkers (mRNA, microRNA, lncRNA and tRFs) offering an alternative and innovative matrix that can be sampling in non-invasive manner. (Step 2) The misregulation of RNA biomarkers will be analyzed by Next-Generation Sequencing (NGS) in order to perform Gene Expression Profile (GEP), revealing their physiological and pathological regulations. (Step 3) The application of artificial intelligence will be crucial in deciphering tear biomarkers misregulation and interactions to guide clinical decision-making for personalized IRD patient management. Minimum RNA input: ≥2 ng total RNA/≥0.3 ng miRNA. Suggested sequencing depth: 5–8 million reads per sample. Anticipated distribution of mapped small RNA: 45–65% miRNAs, 8–15% tRFs, 3–6% lncRNAs. Noise associated with low volume input: a 12–25% increase in mapping loss below the 2 ng threshold. Image adapted from Servier Medical Art (https://smart.servier.com/, accessed on 8 December 2025), licensed under CC BY 4.0 (https://creativecommons.org/licenses/by/4.0/, accessed on 8 December 2025).

MicroRNA tear fluid is a minimally invasive, accessible matrix reflecting ocular and systemic conditions. It allows repeated sampling, enabling longitudinal monitoring. Tears contain nucleic acids, proteins, lipids, and metabolites, providing a wide range of biomarkers (Figure 1).

Tear fluid generally produces 3–10 µL per eye using capillary micro-collection and 0.5–2 µL using Schirmer strips, resulting in an average total RNA recovery of 2–10 ng and miRNA fractions ranging from 0.1 to 0.8 ng. Small RNA sequencing of tears typically yields 1–5 million reads per sample, with 35–55% accurately mapping to identified miRNAs and 5–12% to additional non-coding RNA species (including tRFs and lncRNAs), contingent upon the performance of the library kit and the method of collection.

Schirmer-based sampling results in a 35–60% variability in RNA yield due to dilution from reflex tearing, while micro-capillary tubes generally decrease variability to 10–18%. In comparison studies, Schirmer strips yielded less than 5 ng of total RNA in 55–70% of samples, whereas micro-capillary techniques achieved 6–12 ng in 78–85% of collections.

Although much of the current tear-fluid literature has focused on more common ocular surface and retinal vascular disorders, these conditions provide well-characterized biological settings in which to study molecular pathways—such as inflammation, epithelial stress, angiogenesis, and neurodegeneration—that are also activated in the secondary phases of many rare inherited retinal diseases. As a result, tear-based proteomic and RNA signatures identified in these prevalent conditions offer a valuable translational framework for exploring analogous biomarkers in IRDs, even before direct evidence becomes available in rare genetic cohorts. Their non-invasive nature aligns with precision medicine in IRDs. Proteomic profiling has identified signatures in dry eye, Sjögren’s syndrome, keratoconus, and high myopia [6,20,21]. These findings support tear proteomics as a complementary tool for IRDs. Tear RNA biomarkers, including miRNAs and lncRNAs, are increasingly studied. Tear miRNA profiles can predict anti-VEGF therapy response (e.g., diabetic macular edema) and regulate corneal epithelial homeostasis. Dysregulated tear miRNAs have also been reported in autoimmune ocular diseases. While not yet validated in IRDs, the foundation for tear-omics applications is strong. Challenges remain: variable sampling methods introduce bias, small sample volumes limit yield, and biological variability affects reproducibility. Standardized protocols for tear collection, storage, and normalization are urgently needed for clinical translation [20,21].

Tear-omics can be considered as a clinically scalable advancement in molecular ophthalmology, offering access to highly informative biomarkers in a non-invasive way, with great translational potential. This aspect is particularly relevant in the pediatric population and in syndromic patients with inherited retinal dystrophies, who may be unable to undergo invasive sampling regularly.

Tear fluid is a precious reservoir of biomarkers: not only structural proteins and inflammatory mediators but also regulatory RNA entities, including microRNAs, long non-coding RNAs, and tRNA-derived fragments. Those elements can provide information about processes such as retinal degeneration, angiogenesis, and epithelial stress. Distinct tear microRNA profiles can correlate with responsiveness to anti-VEGF therapy in retinal vascular diseases, while specific tRNA-derived fragments, such as tRF-1001 and tRF-22, have been associated with retinal neovascularization and choroidal vasculopathies [22,23]. Tear-omics can be considered as a ‘liquid biopsy’ for inherited retinal dystrophies and other rare ocular genetically determined disorders, facilitating early risk stratification, prediction of potential therapeutic efficacy, and longitudinal monitoring without requiring intraocular sampling.

Unlike conventional serum or aqueous humor profiling, tear fluid allows repeated temporal characterization in individual patients. This longitudinal approach is particularly relevant in rare diseases, which are characterized by naturally small cohort sizes. In these populations, the intra-individual evolution of the condition over time holds greater diagnostic significance than cross-sectional population averages. Consequently, we propose that tear-based biomarker panels may attain clinical significance even in ultra-rare genotypes, which cannot be framed in the standardization of serial sampling.

Several obstacles have hindered the routine clinical implementation of tear-omics in inherited retinal dystrophies. Barriers include discrepancies in sampling techniques (Schirmer strips versus microcapillary tubes), inconsistencies in pre-analytical processing, and insufficient sample sizes that can be an obstacle for subsequent proteomic and RNA-seq workflows. The subsequent critical advancement in translation necessitates standardized operational methods for tear collection, normalization, storage, and sequencing or proteomic processes. This standardization initiative is regarded as a vital unmet necessity and a tangible potential for consensus development within the sector [4].

A wide range of preclinical and experimental models has contributed to clarifying how non-coding RNAs and other tear-accessible biomarkers participate in retinal and choroidal pathology. Although these models do not replicate the primary genetic defect underlying IRDs, they are highly informative because they capture common downstream mechanisms—such as hypoxia-driven angiogenesis, oxidative and metabolic stress, microglial activation, and fibrotic remodeling—that also shape disease progression in many rare genetic disorders. Therefore, regulatory RNA signatures characterized in these settings provide mechanistic clues that may translate to IRD biology and help guide biomarker discovery in genetically defined patient populations.

Several preclinical models have offered mechanistic understanding of the involvement of non-coding RNA species and tear-accessible biomarkers in retinal and choroidal pathology associated with rare ocular genetic disorders. Models of oxygen-induced retinopathy have revealed dynamic alterations in microRNA networks, including miR-30e-3p, miR-335, miR-30b-5p, and members of the let-7 family, which can modulate hypoxia-induced neovascularization and inflammatory events in both the retina and choroid [15]. This is pertinent in consideration of the association between pathological retinal neovascularization and metabolic imbalance in certain inherited retinal dystrophies.

Experimental research on tRNA-derived fragments has demonstrated that tRF-1001 and tRF-22 influence pro-angiogenic and vasculopathic pathways in ocular tissues, whereas tRNA-Cys-5-0007 has simultaneous anti-inflammatory and anti-angiogenic effects [11,12,13,23]. These findings support the evidence that short RNA fragments serve as upstream regulators of microvascular remodeling, gliosis, and tissue survival in the degenerating retinal pigment epithelium–photoreceptors complex. Several categories of short RNAs are detectable in ocular biofluids, making them promising candidates for biomarker creation and targeted control [12,23].

Data obtained from human subretinal fluid collected during retinal detachment surgery gave evidence that certain microRNAs, such as miR-21 and miR-34, correlate with clinical parameters, including the duration of detachment and the risk of proliferative vitreoretinopathy [14]. These observations provide proof-of-principle that extracellular RNA signatures can reflect disease severity and predict fibroproliferative outcomes in vivo. Collectively, these preclinical and perioperative findings suggest that molecular indicators associated with neovascular stress, mitochondrial dysfunction, gliosis, and fibrotic remodeling can be detected through minimally invasive sampling, and that these pathways intersect with those involved in inherited retinal dystrophies and other rare ocular genetic disorders. Molecular understanding derived from experimental models has directly influenced possible biomarkers for tear-omics pipelines and has supported the exploration of targeted RNA-based therapies for prospective clinical translation.

Collectively, microRNAs, long non-coding RNAs, and tRNA-derived fragments define a regulatory network wherein molecules identified through retinal and choroidal NGS studies may be monitored in accessible biofluids like tears, facilitating the dynamic tracking of angiogenesis, neuroinflammation, and extracellular matrix remodeling through the progression of rare inherited diseases.

## 4. Integrating Genomics with Tear-Omics for Precision Ophthalmology

Integrating genomic sequencing with tear-based molecular profiling offers a complementary, multilayered approach to stratifying patients with IRDs. Genomic data establish the causal variants, inheritance patterns, and predicted disease trajectory, whereas tear-omics captures dynamic molecular signatures that reflect ongoing tissue stress, inflammation, angiogenic activity, and treatment response. Together, these modalities enable a shift from static diagnosis toward continuous, individualized disease monitoring capable of informing patient-specific therapeutic decision-making.

Computational tools, particularly machine-learning and multimodal data-integration pipelines, have shown promise in correlating molecular signatures with structural and functional outcomes such as optical coherence tomography (OCT), OCT-angiography metrics, and visual function scores. Early studies have demonstrated the feasibility of combining genetic data with tear-fluid proteomic or miRNA profiles to identify biomarker patterns predictive of anti-VEGF responsiveness or progression of retinal vascular disease [22]. For a patient with a confirmed IRD pathogenic variant (Step 1: genomic testing), tear-fluid small RNA sequencing (Step 2) facilitates the serial quantification of angiogenic, hypoxia-related, or microglial-associated non-coding RNAs. Data integration transpires at Step 3, wherein genotype (e.g., *ABCA4*, *RHO*), penetrance-modifying polymorphisms, and structural OCT progression scores are examined in conjunction with longitudinal ncRNA trajectories. Step 4 facilitates risk indexing; for instance, a 3–5-fold increase in miR-21 or miR-146a prior to ellipsoid-zone thinning by 4–9 months. Step 5 delineates the frequency of surveillance and the stratification of trial eligibility.

In treatment-stratified cohorts, miR-21, miR-146a, and miR-155 exhibit predictive capability for anti-VEGF responsiveness, with an AUC of 0.72–0.84, sensitivity of 68–81%, and specificity of 66–79%. Conversely, miR-200c and miR-195 are associated with poor responders, displaying over 4.5-fold increases in non-responders compared to less than 2-fold changes in responsive profiles.

However, model interpretability and reproducibility remain essential for clinical adoption. Standardization of pre-analytical variables—including tear collection methods, RNA/protein extraction workflows, and normalization strategies—represents a critical unmet need.

From a translational perspective, numerous ncRNAs have already surfaced as candidates linking tissue-level NGS data with tear-fluid signatures across varying stages of disease severity. Retinal transcriptomic investigations in ischemic and neovascular models have consistently demonstrated the upregulation of miR-21, miR-155, and members of the miR-17/92 cluster during hypoxia-induced angiogenesis and microglial activation. Concurrently, tear-fluid profiling in diabetic retinopathy, dry eye disease, and autoimmune keratopathy has revealed corresponding elevations in the same or closely related miRNAs across clinically defined severity levels. Likewise, miR-146a and members of the miR-200 family have exhibited increasing dysregulation in retinal tissue and tears, correlated with chronic inflammation, epithelial stress, and barrier dysfunction. Despite the absence of systematic mapping of longitudinal ncRNA time courses in genetically confirmed inherited retinal dystrophies, cross-disease observations propose a model wherein a static genomic diagnosis identifies the causal variant, while ncRNA signatures, observable in both retinal NGS datasets and tear fluid, fluctuate in response to the activation of ischemic, inflammatory, or fibrotic pathways during the progression of the rare disease. In this context, critical time points, including the shift from quiescent to exudative neovascularization, the emergence of clinically observable macular edema, and the rapid progression of outer-retinal atrophy, can be regarded as inflection points where ncRNA trajectories in tears deviate from baseline, offering clinically relevant insights.

From an operational standpoint, the incorporation of genomics and tear-omics into standard care seems attainable within tertiary centers that already conduct NGS for inherited retinal diseases. Genomic testing can be conducted once, at baseline, utilizing established clinical protocols and counseling frameworks. Tear sampling necessitates solely non-invasive collection methods, such as capillary tubes or Schirmer strips, and can be synchronized with scheduled appointments, with proteome or small RNA profiling conducted in batches utilizing established mass spectrometry or sequencing platforms. Turnaround periods of many weeks are suitable for chronic disease monitoring rather than for acute decision-making. Clinical evidence from pilot studies on diabetic macular edema and neovascular age-related macular degeneration has shown that tear miRNA signatures can differentiate responders to anti-VEGF treatment, suggesting that fluid-based ncRNA profiling can guide therapeutic decisions in retinal vascular diseases. As of now, there have been no known registered interventional trials that prospectively integrate genetic stratification with tear-fluid omics specifically for rare inherited retinal or optic nerve illnesses, and current efforts have been limited to observational or exploratory studies. The demonstrated conceptual and technical feasibility in these prevalent conditions establishes a robust basis for future trials in genetically defined rare ocular cohorts, where tear-omics may be utilized as a secondary endpoint or as a mechanism for monitoring molecular responses.

Future precision-ophthalmology workflows may incorporate genomics at baseline for diagnostic confirmation and variant interpretation, followed by tear-omics for longitudinal monitoring through repeated, minimally invasive sampling. Within such a framework, serial tear profiling could support early risk stratification, detection of molecular deterioration before structural decline, evaluation of gene-therapy efficacy, and generation of individualized “digital twins” integrating genetic, molecular, imaging, and clinical data. Table 2 provides an overview of how NGS, long-read sequencing, and tear-omics serve as complementary pillars within this integrated model.

A coherent conceptual framework for integrating genomics with tear-based molecular profiling in retinal disease can be articulated by considering how these modalities capture different but complementary layers of biological information. Genomic sequencing provides the foundational, static layer that defines the inherited architecture of disease, identifies causative variants, clarifies inheritance patterns, and anchors the patient within a predictable biological trajectory. This baseline remains constant throughout life and establishes the molecular constraints within which disease development and therapeutic responsiveness occur.

Tear fluid contributes a second, dynamic layer that reflects the ongoing molecular activity of ocular tissues. Changes in tear-derived ncRNAs, proteins, and extracellular vesicle cargo mirror fluctuations in inflammation, angiogenesis, oxidative stress, and barrier integrity, offering a real-time view of disease activity. Whereas genomic data describe what the disease is, tear-omics conveys what the disease is doing at any given moment. The interplay between these static and dynamic dimensions creates a multidimensional understanding of pathophysiology, where inherited predisposition can be interpreted in the context of current biological state.

A third element of this framework arises from the integration of molecular information with structural and functional markers obtained through clinical imaging and visual performance metrics. Computational approaches capable of synthesizing these datasets allow molecular signatures to be interpreted alongside OCT features, vascular parameters, or visual function, thereby linking biochemical changes to measurable tissue outcomes. Such integrated analyses support the identification of biomarker patterns associated with disease progression or treatment responsiveness and encourage the development of more interpretable, clinically grounded predictive models.

In various vascular and degenerative models, deviations in tear-miRNA (notably miR-146a, miR-21, and tRF-22) have occurred 4–9 months prior to OCT-detected retinal thinning or the onset of neovascularization, indicating a quantifiable temporal precedence of molecular changes over structural deterioration

Within this structure, the combined use of genomics and tear-omics supports a more continuous, individualized approach to retinal disease management. Genomic testing serves as the anchor for diagnostic confirmation and therapeutic eligibility, while repeated tear-fluid sampling enables ongoing surveillance of disease activity, early recognition of molecular deterioration, and evaluation of therapeutic efficacy, including gene-based interventions. Over time, the accumulation of these layered datasets—genetic, molecular, structural, and clinical—creates the foundation for highly personalized models of disease behavior capable of guiding treatment decisions with far greater precision than any single modality can achieve.

## 5. Limitations, Open Questions and Future Directions

Despite substantial progress, several challenges persist in the management of IRDs. Their extreme genetic heterogeneity—now involving more than 300 genes—continues to complicate genotype–phenotype correlations. Variants of uncertain significance, incomplete penetrance, and variable expressivity can hinder clinical interpretation and genetic counseling. In parallel, tear-omics still faces technical barriers, including sampling variability, low sample volume, and biological heterogeneity. Addressing these issues is essential for establishing robust, clinically meaningful tear-based biomarkers.

The majority of tear-omics cohorts are inadequately powered, with median sample sizes ranging from 14 to 23 participants per study, and merely 11% of published research surpassing n = 40, particularly within genetically defined IRD subsets.

Nevertheless, the convergence of advanced genomic sequencing with tear-fluid molecular profiling offers a compelling path forward. Long-read sequencing is poised to complement standard NGS approaches by refining variant interpretation, improving structural variant detection, and strengthening diagnostic certainty. At the same time, expanding work on non-coding RNAs—including miRNAs, lncRNAs, and tRNA-derived fragments—has begun to identify novel therapeutic targets and molecular signatures relevant to retinal degeneration, angiogenesis, and neuroinflammation. Tear fluid, accessible non-invasively and amenable to repeated sampling, provides a unique opportunity to capture these dynamic molecular changes and to monitor disease activity or treatment response longitudinally.

In synthesis, this review highlights a key gap in the current literature: while genomic characterization provides the essential foundation for diagnosing IRDs, functional monitoring remains largely descriptive, episodic, and insufficiently sensitive to early or subclinical changes. Integrating genomic data with tear-omics could shift IRDs from static diagnostic labels to molecularly trackable conditions in which disease activity, progression, and therapeutic response can be measured over time.

Importantly, tear fluid should no longer be regarded solely as an indicator of anterior segment disease—such as dry eye, keratoconus, or autoimmune keratopathy—but as a biologically informative matrix that can reflect retinal and choroidal processes through RNA and protein signatures associated with angiogenic signaling, neurodegeneration, oxidative stress, and microglial activation. When combined with high-resolution genomic data, including long-read sequencing for structural variant resolution, these tear-derived biomarkers could enable the development of individualized predictive models, even within the constraints of the small patient cohorts characteristic of rare diseases.

However, major limitations remain. Most studies in this field are single-center and underpowered, limiting generalizability [20,21]. There is a pressing need for coordinated multicenter initiatives to validate tear-based biomarkers alongside genomic variant interpretation, to harmonize pre-analytical and analytical protocols, and to develop standardized operating procedures for tear collection, processing, and sequencing. Future priorities include prospective trials evaluating clinical utility, harmonization of workflows, and AI-supported integration of multi-omics, imaging, and functional data.

If these challenges are addressed, the combined application of genomics and tear-omics has the potential to transform clinical management of rare ocular genetic diseases, enabling accessible, cost-effective, and truly personalized ophthalmic care.

## Figures and Tables

**Table 2:** Comparative Overview of Genomic and Tear-omics Approaches.

Approach	Strengths	Limitations	Applications in IRDs
Next-Generation Sequencing (NGS)	High throughput; cost-effective; detects SNVs and small indels; broad panels available	Limited for structural variants and phasing; variants of uncertain significance (VUS) common	Routine diagnosis; gene discovery; genotype–phenotype correlation studies
Long-Read Sequencing (LRS)	Resolves phasing; detects structural variants and repetitive regions; better variant interpretation	Higher cost; lower throughput; bioinformatics pipelines less standardized	Reclassification of VUS; improved diagnostic yield in unsolved IRDs; discovery of novel pathogenic mechanisms
Tear-omics (proteomics and ncRNA)	Non-invasive; allows longitudinal monitoring; reflects local ocular pathophysiology	Low sample volume; variability in collection methods; pre-analytical standardization needed	Biomarker discovery; monitoring treatment response; potential integration with genomics for precision medicine

## Data Availability

No new data were created or analyzed in this study. Data sharing is not applicable to this article.
